# Performance-Based Cognitive Screening Instruments: An Extended Analysis of the Time *versus* Accuracy Trade-off

**DOI:** 10.3390/diagnostics5040504

**Published:** 2015-11-27

**Authors:** Andrew J. Larner

**Affiliations:** Cognitive Function Clinic, Walton Centre for Neurology and Neurosurgery, Lower Lane, Fazakerley, Liverpool L9 7LJ, UK; E-Mail: a.larner@thewaltoncentre.nhs.uk; Tel.: +44-1515-298113

**Keywords:** diagnosis, cognitive screening instruments, dementia, accuracy

## Abstract

Early and accurate diagnosis of dementia is key to appropriate treatment and management. Clinical assessment, including the use of cognitive screening instruments, remains integral to the diagnostic process. Many cognitive screening instruments have been described, varying in length and hence administration time, but it is not known whether longer tests offer greater diagnostic accuracy than shorter tests. Data from several pragmatic diagnostic test accuracy studies examining various cognitive screening instruments in a secondary care setting were analysed to correlate measures of test diagnostic accuracy and test duration, building on the findings of a preliminary study. High correlations which were statistically significant were found between one measure of diagnostic accuracy, area under the receiver operating characteristic curve, and surrogate measures of test duration, namely total test score and total number of test items/questions. Longer cognitive screening instruments may offer greater accuracy for the diagnosis of dementia, an observation which has possible implications for the optimal organisation of dedicated cognitive disorders clinics.

## 1. Introduction

Early diagnosis of cognitive disorders remains largely a clinical exercise, based on history from patient and informant, and examination supplemented by administration of cognitive screening instruments (CSIs), with the reference or criterion standard being judgment of an experienced clinician applying widely accepted diagnostic criteria. The development of robust disease biomarkers may significantly impact on this diagnostic procedure in the future, but until such time as these sophisticated tests become widely available the traditional approach to diagnosis will prevail, meaning that CSIs will remain an integral part of clinical assessment, perhaps increasingly administered in computerised format.

It is over a century since a trade-off between the speed and the accuracy of performance of voluntary movements was first recognised, such that more accurate movements are performed more slowly [1]. As speed is inversely proportional to time, this trade-off may also be formulated as time *versus* accuracy, with longer times being required for greater accuracy.

A previous analysis examined whether this trade-off might be applicable to the diagnostic accuracy of CSIs and their administration time, or in other words whether shorter CSIs were less accurate than longer ones which may sample more cognitive domains. Since the actual duration of test administration is not routinely measured in the clinical setting, more easily accessible surrogate measures of test duration were used, namely the overall test score and the total number of items/questions in the test. Correlations between these measures and overall test diagnostic accuracy (or correct classification accuracy) were found for a selection of commonly used CSIs [2].

The aim of the study presented here was to extend the previous analysis to examine not only correct classification accuracy but also area under the receiver operating characteristic curve, another single or global measure of test diagnostic accuracy, as well as examining another short performance-based cognitive screening instrument, the Mini-Addenbrooke’s Cognitive Examination, not previously subjected to this analysis.

## 2. Materials and Methods

The datasets from several pragmatic prospective diagnostic test accuracy studies [3] were used [4,5,6,7,8,9,10,11] (Table 1), which examined the following (nine) performance-based CSIs: Addenbrooke’s Cognitive Examination (ACE) [12], Addenbrooke’s Cognitive Examination-Revised (ACE-R) [13], DemTect [14], Mini-Addenbrooke’s Cognitive Examination (M-ACE) [15], Mini-Mental Parkinson (MMP) [16] used as a general CSI, Mini-Mental State Examination (MMSE) [17], Montreal Cognitive Assessment (MoCA) [18], Six-item Cognitive Impairment Test (6CIT) [19], and the Test Your Memory (TYM) test [20].

These pragmatic studies followed a standardized format [21] of cross-sectional assessment of consecutive outpatient referrals with some or all of the following elements: semi-structured patient history enquiring about cognitive symptoms and functional performance, with collateral history where possible; administration of CSIs; neuroradiological examination (CT all patients; interval MRI in some cases); and formal neuropsychological assessment in some cases. Standard diagnostic criteria for dementia (DSM-IV) and mild cognitive impairment (MCI) were used. Reference standard was the judgment of an experienced clinician applying widely accepted diagnostic criteria, but blind to CSI scores in order to avoid review bias [21].

Diagnostic accuracy for dementia was defined for each CSI in two ways. Correct classification accuracy was calculated as the sum of true positives and true negatives divided by the total number of patients tested. Area under the receiver operating characteristic curve (AUC ROC) was calculated in the standard manner from the ROC curve plotting false positive rate on the x axis (abscissa) against sensitivity (“hit rate”) on the y axis (ordinate) [3].

**Table 1:** Study demographics.

CSI	Setting	*n*	Dementia Prevalence	M:F (% Male)	Age Range (Years)	Ref.
ACE	CFC	285	49%	147:138 (52)	N/A	[4]
ACE-R	CFC	243	35%	135:108 (56)	24–85 (mean 60)	[5]
DemTect	CFC	111	52%	52:59 (47)	23–86 (median 63)	[6]
M-ACE	CFC	135	18%	71:64 (53)	18–88(median 60)	[7]
MMP, MMSE	CFC	225	21%	130:95 (58)	20–86 (median 62)	[8]
MoCA	CFC	150	24%	93:57 (62)	20–87 (median 61)	[9]
6CIT	CFC	245	20%	124:121 (51)	16–94 (median 59)	[10]
TYM	CFC and Old Age Psychiatry memory clinic	224	35%	130:94 (58)	20–90 (mean 63)	[11]

Abbreviations: CSI = Cognitive Screening Instrument; CFC = Cognitive Function Clinic; ACE = Addenbrooke’s Cognitive Examination; ACE-R = Addenbrooke’s Cognitive Examination-Revised; (ACE-R); M-ACE = Mini-Addenbrooke’s Cognitive Examination; MMP = Mini-Mental Parkinson; MMSE = Mini-Mental State Examination; MoCA = Montreal Cognitive Assessment; 6CIT = Six-Item Cognitive Impairment Test; TYM = Test Your Memory (TYM) test.

Measures of test duration (surrogate measures of time) were either the total test score or the total number of test items/questions [2] (Table 2). No data were collected on the actual time taken for test administration, but estimated times collated from the original publications describing each of these tests [12,13,14,15,16,17,18,19,20] are included as a guide (Table 2, column 2).

**Table 2:** CSI approximate administration time and surrogate measures thereof (total test score, total number of test items/questions) with diagnostic accuracy (overall correct classification and area under ROC curve) for diagnosis of dementia.

CSI	Approximate, Estimated, Administration Time (Minutes, (Ref.))	Total Test Score	Number of Test Items or Questions	Overall Correct Classification (95% CI)	Area under ROC Curve (95% CI)	Ref.
ACE	15–20, [12]	100	52	0.84 (0.80–0.88)	0.93 (0.90–0.96)	[4]
ACE-R	15–20, [13]	100	66	0.89 (0.85–0.93)	0.94 (0.91–0.97)	[5]
DemTect	8–10, [14]	18	13	0.78 (0.71–0.86)	0.87 (0.80–0.93)	[6]
M–ACE	5–10, [15]	30	10	0.84 (0.78–0.91)	0.86 (0.83–0.90)	[7]
MMP	5–10, [16]	32	23	0.86 (0.81–0.91)	0.89 (0.84–0.94)	[8]
MMSE	5–10, [17]	30	21	0.86 (0.81–0.90)	0.87 (0.81–0.92)	[8]
MoCA	10–15, [18]	30	22	0.81 (0.75–0.88)	0.91 (0.86–0.95)	[9]
6CIT	2–3, [19]	28	7	0.80 (0.75–0.85)	0.90 (0.85–0.95)	[10]
TYM	5–10 (self-administered under medical supervision), [20]	50	25	0.83 (0.78–0.88)	0.89 (0.84–0.93)	[11]

Measures of test accuracy were considered the outputs or effects, hence the dependent variables, plotted on the ordinate (y-axis). Measures of test duration were considered the inputs or causes, hence independent variables, plotted on the abscissa (x-axis). Correlations between these parameters were calculated.

## 3. Results

**Figure 1:**
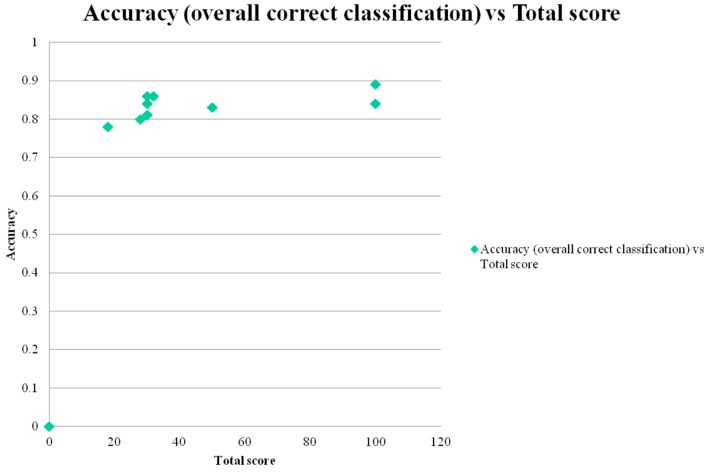
Scatter plot of test correct classification accuracy *versus* total test score (= surrogate measure of test administration time).

**Figure 2:**
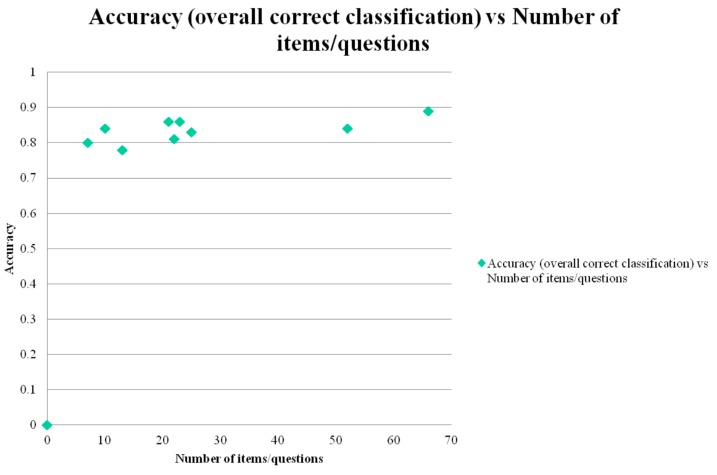
Scatter plot of test correct classification accuracy *versus* total number of test items/questions (= surrogate measure of test administration time).

Correct classification accuracy was positively correlated with both total test score (*r* = 0.58; Figure 1) and with total number of test items/questions (*r* = 0.66; Figure 2). Both correlations were classified as moderate, and respectively did not reach statistical significance (*t* = 1.89, *df* = 7, *p* > 0.1) or showed a trend towards significance (*t* = 2.33, *df* = 7, 0.1 > *p* > 0.05).

**Figure 3:**
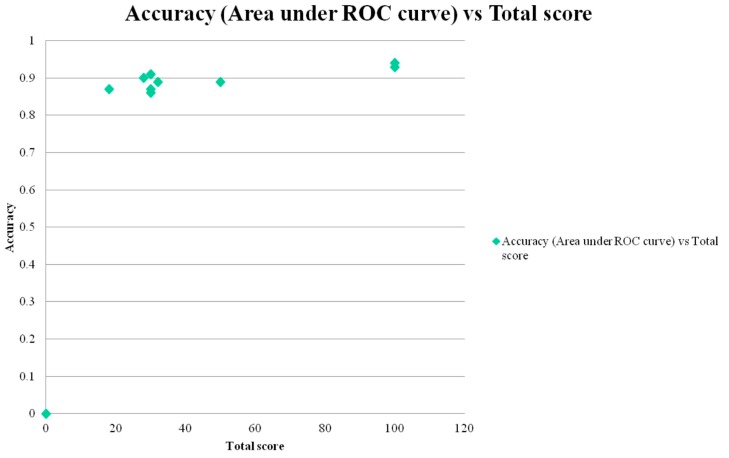
Scatter plot of area under ROC curve *versus* total test score (= surrogate measure of test administration time).

**Figure 4:**
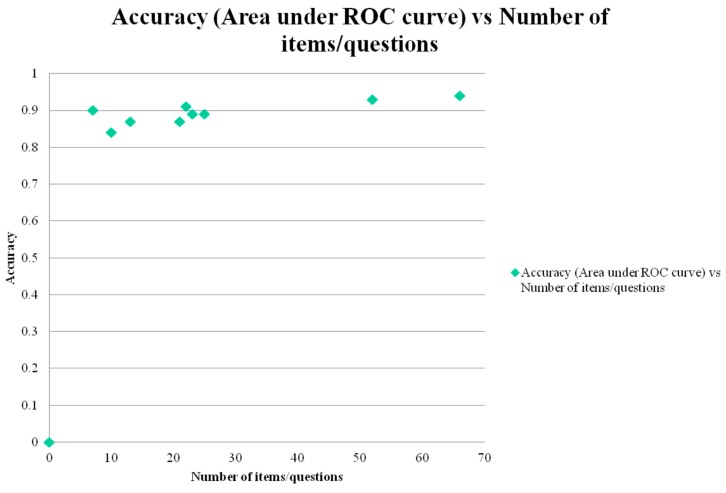
Scatter plot of area under ROC curve *versus* total number of test items/questions (= surrogate measure of test administration time).

AUC ROC curve was positively correlated with total test score (*r* = 0.83; Figure 3) and with total number of test items/questions (*r* = 0.79; Figure 4). Both correlations were classified as high and both reached statistical significance (*t* = 3.86, *df* = 7, *p* < 0.01; and *t* = 3.46, *df* = 7, *p* < 0.02, respectively).

**Figure 5:**
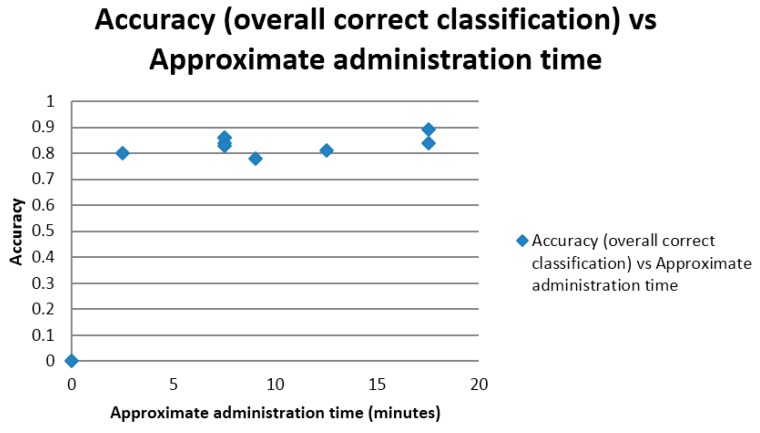
Scatter plot of test correct classification accuracy *versus* approximate test administration time.

**Figure 6:**
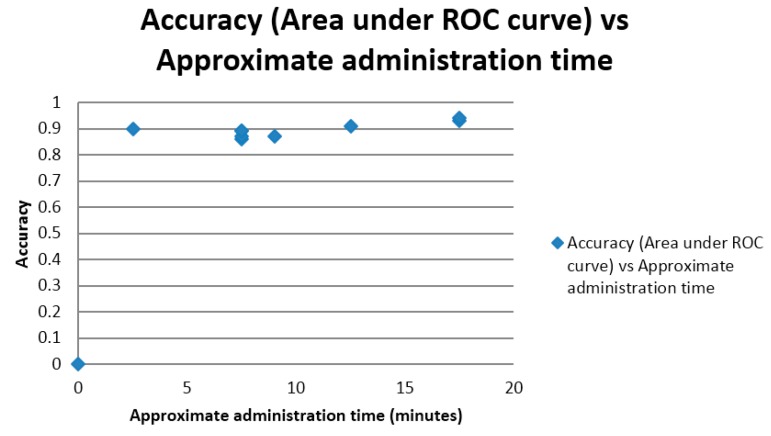
Scatter plot of area under ROC curve *versus* approximate test administration time.

Although no measurements of time were made during the test accuracy studies, estimated times of test administration were correlated with the measures of test accuracy. Both correct classification accuracy and AUC ROC curve were positively correlated with approximate, estimated, test administration time (*r* = 0.43; Figure 5; and *r* = 0.73; Figure 6), correlations which were classified as low and high respectively. The latter reached statistical significance, (*t* = 2.81, *df* = 7, *p* < 0.05), but the former did not (*t* = 1.24, *df* = 7, *p* > 0.1).

## 4. Discussion

This analysis confirms [2] and extends the suggestion that there is a trade-off for CSIs between two surrogate measures of duration of test administration and two measures of test diagnostic accuracy. Investing more time during the clinical encounter in administering longer CSIs might therefore pay dividends in terms of improved accuracy of dementia diagnosis.

Of course there are a number of shortcomings to the approach used in this analysis. Firstly, inter-study comparisons are problematic, notwithstanding the consistency of study protocols [21] and authorship of the studies examined here.

Secondly, the tests examined are screening tests and not diagnostic tests, and have been used in clinic-based populations which are inevitably selected compared to community or population-based samples.

Thirdly, both the unitary measures of diagnostic accuracy examined may be criticised [3]: correct classification accuracy is dependent on disease prevalence and may therefore differ in different populations; AUC ROC combines test accuracy over a range of thresholds which may be both clinically relevant and clinically nonsensical [22]. A cost-benefit or cost-worthiness analysis of screening would determine the benefits of true positive and true negative test results *versus* the costs of false positive and false negative results, and although the requisite analytical tools are available [23,24] this is much more difficult to do, perhaps requiring evaluation of the entire diagnostic test-treatment pathway, a so-called phase IV research question [3].

Fourthly, both the measures of test duration used were surrogates, rather than timed administration, which may vary between patients, dependent in part on degree of cognitive impairment. Other time surrogates, such as number of cognitive domains tested, might also have been examined, although the spread of values for this parameter would be less than for either of the two selected measures. The increased use of computerized testing in the future will allow measurement of actual time of test performance, and hence permit analysis of cost-benefit or cost-worthiness per unit time to define the most efficient test. The analysis using estimated test administration time suggested a correlation with one measure (AUC ROC curve) of test accuracy. No other studies explicitly examining time of test administration and measures of test diagnostic accuracy have been identified.

Fifthly, correlation between measures is not necessarily indicative of causality, although the consistency of the correlations is potentially suggestive. Moreover, statistical significance does not necessarily equate with clinical significance or relevance.

Sixthly, whether the findings also hold true for diagnosis of mild cognitive impairment or for informant-based CSIs, or in community-based or population-based patient samples, has not been examined.

Notwithstanding these limitations, it might be pragmatically argued in light of these findings that the policy of longer outpatient clinic appointments for patients with cognitive complaints (45–60 min), as compared to general neurology outpatient appointments (15–30 min), is justified in order to permit adequate time for the administration of longer CSIs because of the desired outcome of more accurate diagnosis. To borrow informally an analogy from science and engineering, there may be a lower “signal to noise ratio” when using longer CSIs (where the delivered strength of “signal” is related to statistical significance, and “noise” to standard deviation) due to their increased “bandwidth” (*i.e.*, broader range of test scores or items). The greater neuropsychological coverage of longer CSIs, one of the desiderata suggested by expert consensus [25], may reduce test ceiling and floor effects. This may also be quantified by the higher Q* index of longer CSIs, that being the point of indifference in ROC space where sensitivity and specificity are equal, another unitary metric of diagnostic accuracy that may be used to compare CSIs [26].

Aside from these methodological and quantitative issues, clinician preference for specific tests may also need to be factored into the equation of which screening test to use in practice. Speed of administration was one of the factors, along with effectiveness and ease of administration, emerging in one survey documenting specialty clinicians’ preferences [27].

## 5. Conclusions

This study presents evidence of a trade-off for CSIs between measures of duration of test administration and measures of test diagnostic accuracy: longer tests are more accurate. Hence, in the clinical encounter, more time spent administering longer CSIs might improve accuracy of dementia diagnosis.
